# Development of Endothelial-Specific Single Inducible Lentiviral Vectors for Genetic Engineering of Endothelial Progenitor Cells

**DOI:** 10.1038/srep17166

**Published:** 2015-11-27

**Authors:** Guanghua Yang, M. Gabriela Kramer, Veronica Fernandez-Ruiz, Milosz P. Kawa, Xin Huang, Zhongmin Liu, Jesus Prieto, Cheng Qian

**Affiliations:** 1 Division of Gene Therapy and Hepatology, Center for Applied Medical Research (CIMA), University of Navarra, Pamplona, Spain; 2 Research Center for Translational Medicine, East Hospital, School of Medicine, Tongji University, Shanghai 200120, China; 3 Magee-Womens Research Institute, University of Pittsburgh School of Medicine, Pittsburgh, PA 15213, USA

## Abstract

Endothelial progenitor cells (EPC) are able to migrate to tumor vasculature. These cells, if genetically modified, can be used as vehicles to deliver toxic material to, or express anticancer proteins in tumor. To test this hypothesis, we developed several single, endothelial-specific, and doxycycline-inducible self-inactivating (SIN) lentiviral vectors. Two distinct expression cassettes were inserted into a SIN-vector: one controlled by an endothelial lineage-specific, murine vascular endothelial cadherin (mVEcad) promoter for the expression of a transactivator, rtTA2S-M2; and the other driven by an inducible promoter, TREalb, for a firefly luciferase reporter gene. We compared the expression levels of luciferase in different vector constructs, containing either the same or opposite orientation with respect to the vector sequence. The results showed that the vector with these two expression cassettes placed in opposite directions was optimal, characterized by a robust induction of the transgene expression (17.7- to 73-fold) in the presence of doxycycline in several endothelial cell lines, but without leakiness when uninduced. In conclusion, an endothelial lineage-specific single inducible SIN lentiviral vector has been developed. Such a lentiviral vector can be used to endow endothelial progenitor cells with anti-tumor properties.

For gene therapy-based anti-tumor treatment, therapeutic genes need to be specifically introduced and highly expressed in neoplastic cells, which remains a challenge in the field. Although some lentiviral vectors and replication deficient recombinant adenovirus vectors carrying specific transgenes demonstrate clear therapeutic benefits in a variety of animal tumor models, clinical trials show that these gene therapy systems possess very low anti-tumor capability because of their low specificity in the transduction of neoplastic cells^1^. An alternative strategy has been developed for gene therapy of solid tumors, based on the observation that tumor growth depends on the number of recruited endothelial cells, which contribute to the generation of functional neo-vasculature. Endothelial progenitor cells (EPCs) are considered functional platforms for gene therapy because of their ability to home to the tumor vasculature and to develop new vessels. Bone marrow–derived EPCs have also been frequently detected both in the circulation of cancer patients and in lymphoma-bearing mice. In addition, tumor-targeted migration of EPC from the bone marrow is correlated with tumor volume and the production of VEGF by tumor cells^2^,^3^. The homing of EPCs to the tumor vasculature may lead to their incorporation throughout the tumor mass — up to 95% of the tumor vasculature in the peripheral region^4^,^5^. Transduction of these endothelial cells with therapeutic genes holds the potential to retard the tumor growth—even to eradicate it.

Lentiviral vectors are unique tools for gene delivery into the hematopoietic system because of their biological properties and the relatively easy manipulations required for *ex vivo* gene transfer^1^. In addition to differentiated cells, lentiviral vectors can efficiently transduce committed progenitors and primitive hematopoietic stem cells^6^,^7^. One study has shown that lentiviral vectors can be used for the *in vitro* transduction of human umbilical vein endothelial cells (Huvec) and human bone marrow–derived mesenchymal stem cells with high efficiency^8^. The angiogenic potential of EPCs genetically modified by lentiviruses may be particularly useful in anti-angiogenic therapies of cancer; e.g., in attenuated tumor growth, induced tumor apoptosis and increased survival *in vivo* and *in vitro*. It has been proposed that delivering these genes by EPC has the advantage of generating high concentration of proteins that is specific to tumors, avoiding systemic toxicity. However, safety also requires that the expression of the therapeutic genes to be well controlled. For selective gene expression in endothelial cells, endothelial cell–specific promoters are required^9^. Of note, the vascular endothelial cadherin (VEcad) gene is exclusively and constitutively expressed in endothelial cells^9^ and may be a good candidate for genetic manipulation to achieve specific destruction of solid tumor vascular system.

For controlled gene expression *in vivo*, the Tet-on/off system has been widely used^10^. In Tet-on/off system, a reverse repressor of tetracycline operon, rtetR, is fused to a herpes simplex virus transcriptional factor VP16, to generate a reverse tetracycline-controlled transactivator (rtTA), which interacts with the inducible promoter in the presence of doxycycline (Dox) and activates transcription^1^,^10^,^11^. The inducible The tetracycline-responsive element (TRE) promoter is composed of seven copies of the Tet operator (tetO) fused to a cytomegalovirus minimal promoter region (CMVm). For better performance, one mutant derived from rtTA, named rtTA2S-M2, was introduced into the Tet-on/off system. This mutant transactivator binds with much lower efficiency to the tetO regions than rtTA in the uninduced state, and its VP16 domain was shortened to avoid cell toxicity^11^,^12^. This mutant transactivator is highly sensitive to Dox; it can induce the same gene expression levels at 10% of Dox dose required with the original rtTA^12^. The fused CMVm promoter, due to its self-activation, may cause a basal activation of the Tet-on system^13^; i.e., expression leakage. Thus, a tTS(Kid) repressor was used in the all-in-one inducible lentiviral vector containing CMVm inducible promoter^14^. Although the repressor protein had no effect on the already minimal uninduced expression level, it had a negative influence on the induction process in the presence of Dox^14^,^15^. To further minimize expression leakage without using repressor molecules in the Tet-on system, a Tet-responsive promoter for albumin (TREalb) was generated^10^.

Tet-regulated transgene expression in a host cell requires the delivery of two expression cassettes, one cassette contains the transactivator gene, and the other contains the transgene of interest^1^,^10^,^11^. However, combining the two expression cassettes (Tet-on system) in a single vector can reduce the potential integration sites in the host cells. The quantity of the vector administered to the patient should also be as small as possible in order to minimize the risk of oncogene activation by the integration of the vector to the host genome^16^. In the case of lentiviral vectors, which belong to the RNA vectors family, the possibilities of inserting more than one expression cassette appear to be limited because of the generic use of the termination of transcription by poly (A) sequences. For the expression of small regulable transgenes, such as green fluorescent protein (GFP), an overlapping expression cassette, placed in the same orientation, is used to minimize the size of the inserted gene^17^. Alternatively, opposite cassette orientations with respect to the vector RNA can also be used in lentiviral vectors in which a monodirectional poly (A) signal has been added to the antisense cassette^14^. The cassette placed in the opposite orientation has shown a higher transgene expression level^14^.

In this study, we developed single endothelial-specific and Dox-inducible self-inactivating (SIN) lentiviral vectors, which was constructed by deleting 133 bp promoter and enhancer elements from the U3 region of the 3′ long terminal repeat (LTR)^18^. The deletion is transferred to the 5′ LTR after reverse transcription and integration in infected cells, resulting in the transcriptional inactivation of the LTR in the proviruses and thus, the risk of oncogene activation due to viral insertional mutagenesis can be eliminated using this safety feature of the SIN lentiviral vectors. We compared the expression level of luciferase as a report gene in different vector constructs in endothelial cell lines. Our goal was to generate a single inducible lentiviral vector for specific transduction of endothelial cells. This vector should have three optimal features: (1) no leakiness of the transgene expression in the uninduced state, (2) a high level of gene expression in the presence of doxycycline as an inducer in the Tet-on system, and (3) a high titer of lentiviral vector production. Our results demonstrated a single endothelial lineage-specific inducible SIN lentiviral vector with opposite orientation of two expression cassettes that meets the above requirements. This lentiviral vector was then used to genetically modify endothelial progenitor cells, endowing them with anti-tumor properties.

## Results

### Construction of inducible lentiviral vectors for endothelial-specific regulable gene expression

**Figure 1:**
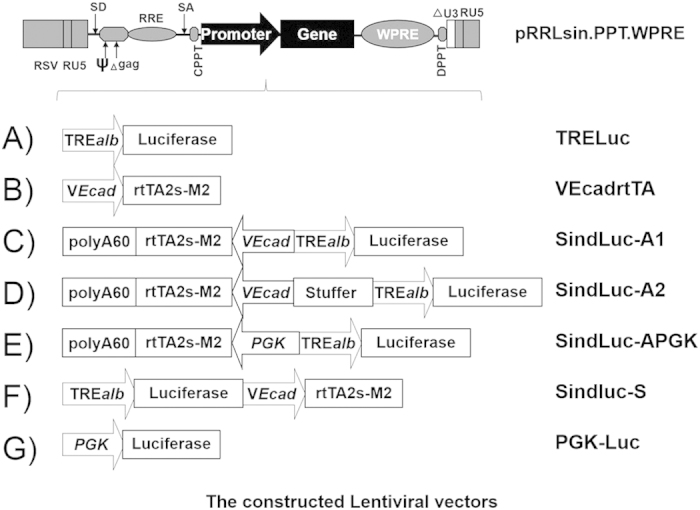
The schematic representations of the lentiviral vectors designed for this study. Vectors are based on the third generation self-inactivated lentiviral vector backbone (pRRLsin.cPPT.WPRE). (**A**) TRE-luc: vector containing luciferase as a reporter gene under the control of the Tetracycline-regulated albumin promoter (TRE/alb). (**B**) VEcadrtTA: a vector containing an endothelial-specific promoter (VEcad) for driving the expression of the Tet-responsive transactivator (rtTA2s-M2). (**C**) SindLuc-A1: a vector containing two cassettes oriented in the antisense direction with respect to the vector RNA. (**D**) SindLuc-A2: a vector containing the stuffer DNA sequence between the two promoters (based on SindLuc-A1). (**E**) SindLuc-APGK: a vector containing human phosphoglycerate kinase (hPGK) promoter replacing VEcad promoter in the SindLuc-A1 vector. (**F**) SindLuc-S: a vector containing two cassettes oriented in the sense direction with respect to the vector RNA sequence. (**G**) PGK-Luc: vector containing human phosphoglycerate kinase (hPGK) promoter driving luciferase gene expression and used as a positive control in the experiments. Abbreviations: RSV, the Rous Sarcoma Virus promoter driving viral mRNA; SD, major splice donor site; ψ, encapsidation signal including the 5′ portion of the gag gene (Δ gag); RRE, Rev-response element; SA, splice acceptor site; cPPT, central polypurine tract; WPRE, the post-transcriptional regulatory element of woodchuck hepatitis virus.

We constructed several lentiviral vectors based on a pRRLsin-derived self-inactivating backbone^19^, in which the different elements of the Tet-on system were introduced sequentially (Fig. 1). Two expression cassettes were inserted into the lentiviral vector. Firefly luciferase was used as a reporter gene under the control of TRE/alb, consisting of a TRE and mouse albumin promoter in the first cassette (vector: TRELuc, Fig. 1A). The second cassette consisted of the endothelial specific promoter (VEcad) for driving the expression of the Tet-responsive transactivator (rtTA2s-M2) in the endothelial cells (vector: VEcadrtTA, Fig. 1B). The second cassette was oriented in the antisense direction with respect to the vector RNA sequence. A monodirectional poly (A) (60PA) sequence was inserted into the second cassette for polyadenylation of the rtTA2s-M2 transcript without disturbing the transcription of the genome during the process of the lentiviral vector production (vector: SindLuc-A1, Fig. 1C). The promoters of the two cassettes were separated by a 2.9-Kb stuffer sequence to suppress the transactivation effects between them (vector: SindLuc-A2, Fig. 1D). In the other construction, where the two cassettes were oriented in the sense direction with respect to the vector RNA, the two cassettes shared the same poly (A) signal of the vector in the 3′long terminal repeat (LTR) (vector: SindLuc-S, Fig. 1F).

### Comparison of production titer of different inducible vectors

In order to compare the production titer of different inducible vectors in Fig. 1, we examined the lentiviral titers in three independent viral production experiments. The lentiviral production was performed on five 15-cm dishes each time, concentrated by ultracentrifugation, and titrated by qPCR. The results indicated that the viral titer was not affected by inserting the antisense expression cassette and 60PA in the lentiviral vector (compared to the SindLuc-S vector) (Table 1). In addition, there is no significant difference between those single inducible vectors containing opposite expression cassette.

### Efficacy of transgene expression mediated by inducible vectors in endothelial and non-endothelial cell lines

**Figure 2:**
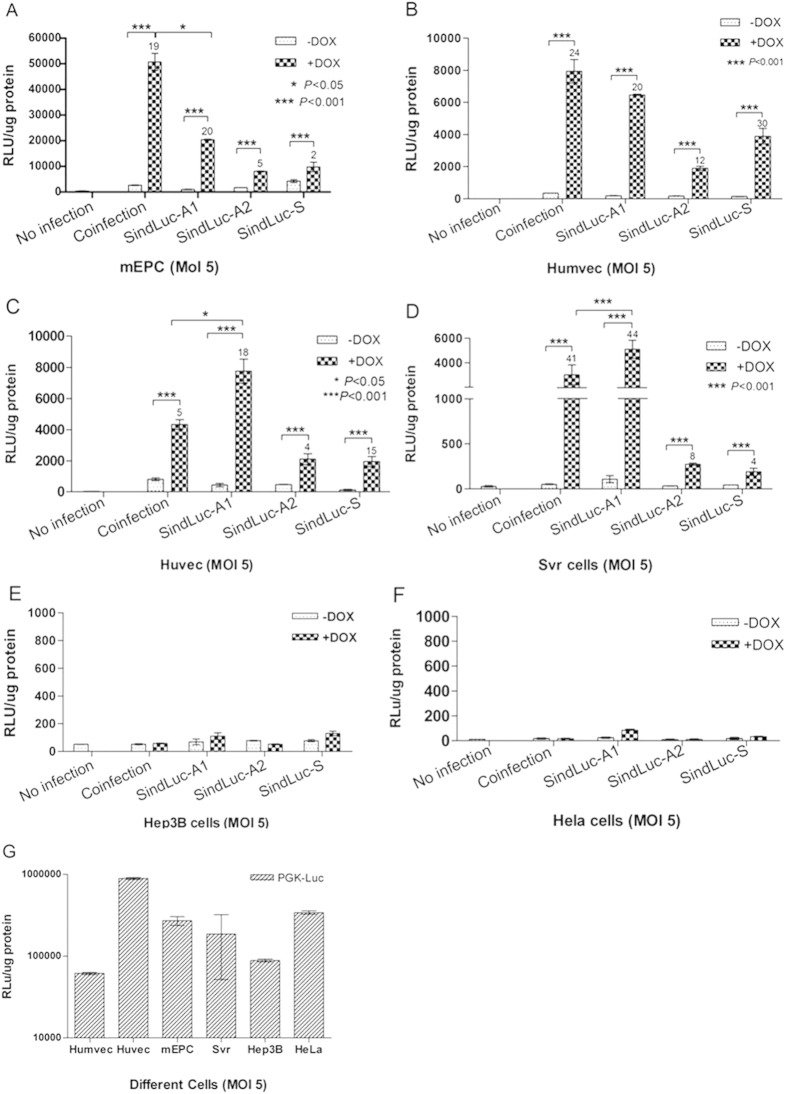
Quantification of doxycycline-dependent gene expression in endothelial cells and non-endothelial cells infected by the different lentiviral vectors encoding luciferase transgene. Cells were transduced (MOI 5) and grown in the presence or absence of Dox (1 μg/mL). Coinfection: TRELuc and VEcadrtTA vectors were used. Luciferase activity was measured 72 hours post-transduction. Fold induction is indicated at the top of each bar. (**A**) Luciferase detection in murine endothelial progenitor cells (mEPC). (**B**) Luciferase detection in human microvascular endothelial cells (Humvec). (**C**) Luciferase detection in human umbilical vein endothelial cells (Huvec). (**D**) Luciferase detection in SVR cells. (**E**) Luciferase detection in Hep3B cells. (**F**) Luciferase detection in HeLa cells. (**G**) Luciferase detection in different cell lines infected with PGK-Luc vector as a positive control. (*P < 0.05; ***P < 0.001). Results are presented as mean ± SD of data from each group, n = 3.

The induction levels of different inducible vectors were analyzed in six endothelial and non-endothelial cell lines, including Humevc, Huvec, mEPC, Svr, Hep3B, and HeLa cells. Each cell line was transduced with different inducible lentiviral vectors at a multiplicity of infection (MOI) of 5, induced with 1 μg/mL Dox for another 68 hours, and harvested for luciferase activity measurement (Fig. 2A–D). We detected basal luciferase expression in the case of the regulable cassette vector, TRELuc, infected together with transactivator expression cassette vector, VEcadrtTA, indicating a high leakage level of luciferase (50–2600 RLU/μg protein) in the absence of Dox. In the case of a single inducible vector, SindLuc-A1, the leakage level of luciferase expression was relatively low (50–1000 RLU/μg protein). Moreover, we observed a high leakage level of luciferase expression (60–4000 RLU/μg protein) using the SindLuc-S vector. A strong luciferase expression was observed when the rtTA2S-M2 transactivator was included in the expressing cassettes (vectors: SindLuc-A1, SindLuc-A2 and SindLuc-S). The activity of the reporter gene in the examined vectors varied, depending on the relative orientation of the transgene- and rtTA2s-M2-containing cassettes. Although the expression level of the same vector in the presence of Dox varied among the cell lines, opposite orientation of the cassettes (vector: SindLuc-A1) resulted in higher expression than did identical orientation (vector: SindLuc-S) in the same cell lines. At the same time, gene induction was observed to be more potent in both Huvec and SVR cells transduced with SindLuc-A1 than in the cells cotransfected with TRELuc and VEcadrtTA (Fig. 2C,D). Insertion of a 2.9-Kb stuffer DNA sequence between the two promoters VEcad and TREalb had a negative effect on the induction in the presence of Dox (Fig. 2A–D). In the non-endothelial cell lines, only a basal level of luciferase expression (8–78 RLU/μg protein) was detected. The expression level was similar to that detected in the cells without infection which served as negative control. Moreover, a PGK-Luc vector was used as a positive control to test the infectivity of different cell lines in all the experiments. Huvec and HeLa cells presented higher accessibility to lentiviral vector infection compared with the other cell lines (Fig. 2G). Taken together, the SindLuc-A1 vector was considered as an optimal construction because of its minimal leakage in the uninduced state and its higher level of transgene induction in the presence of Dox among all the vectors compared in this study.

### Dose-dependent dox-induced transgene expression

**Figure 3:**
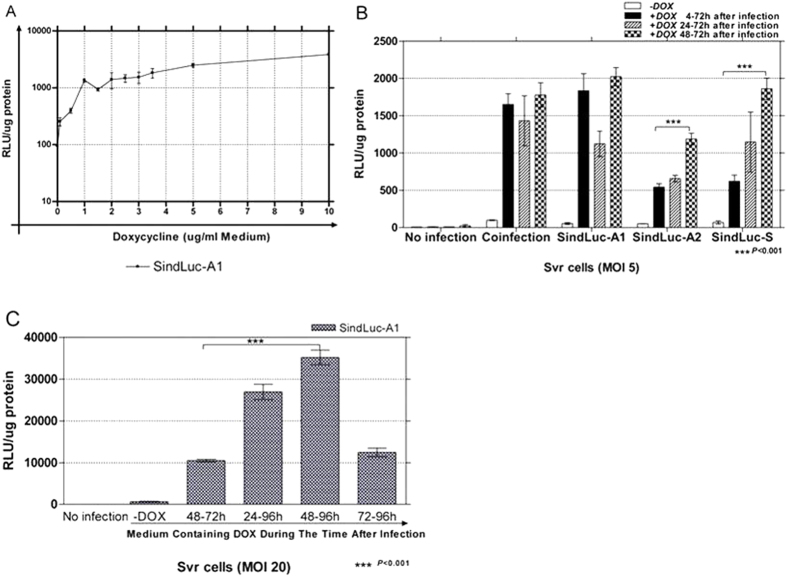
Quantification of doxycycline dose-dependent gene expression in SVR cells infected with SindLuc-A1 vector encoding luciferase transgene controlled by an inducible system. (**A**) SVR cells transduced with SindLuc-A1 vector (MOI 5) and cultured for 68 hours with different concentrations of Dox (0.01; 0.5; 1; 1.5; 2; 2.5; 3; 3.5; 5; 10 μg/mL) were subjected to luciferase activity measurement. (**B**) SVR cells transduced with all the designed inducible vectors (MOI 5) and cultured in the presence of Dox (4 μg/mL) at different time points (4, 24 or 48 hours after infection) were subjected to luciferase activity measurement at 72 hours post transduction. Coinfection: TRELuc and VEcadrtTA vectors were used. (**C**) SVR cells transduced with SindLuc-A1 vector (MOI 5 or 20) and cultured with Dox (4 μg/mL) added to the culture at different time points (4, 24 or 48 hours after infection) were subjected to luciferase activity measurement at 72 or 96 hours post transduction. (***P < 0.001). Results are presented as mean ± SD of data from each group, n = 3.

Tet-on is a doxycycline-dependent system^11^. SVR cells, which are human neoplastic endothelial cells, were transduced with SindLuc-A1 vector (MOI 5) and cultured for 68 hours with various concentrations of Dox (ranging from 0 to 10 μg/mL) before being harvested for luciferase activity measurement. The maximal transgene activation can be reached with 4 μg/mL or higher concentrations of Dox (Fig. 3A), agreeing with published results^14^,^20^. Therefore, an optimal concentration of Dox (4 μg/mL) was selected for all the subsequent experiments.

To optimize the duration of Dox induction, we infected SVR cells with the inducible vectors (MOI 5) and added Dox (4 μg/mL) to the culture medium at different time points (4, 24 or 48 hours after infection). The cells were harvested for measurement of luciferase activity 72 hours after transduction. The highest luciferase expression was observed when the Dox was added at 48 hours after lentivirus infection (Fig. 3B). Coinfection of SVR cells with the vectors encoding the luciferase-inducible expression cassette (TRELuc) and transactivator expression cassette (VEcadrtTA) showed no significant differences in luciferase expression among the various Dox-induction time points tested. Infection with the SindLuc-A1 vector reached the highest induction level at the presence of Dox for 4 or 48 hours after infection. However, both SindLuc-A2 and SindLuc-S vectors achieved the highest luciferase expression with 48 hours of Dox induction following lentiviral infection.

To determine whether longer Dox induction could further enhance transgene expression, we compared the 24-, 48-, and 72-hour Dox induction of infected endothelial cells. When the SVR cells were infected with the single inducible vector SindLuc-A1 at MOI 20 and harvested for luciferase expression measurement at 72 hours or 96 hours post transduction, we observed the highest luciferase expression when cells were harvested 96 hours post transduction with 48 hours of Dox induction (Fig. 3C).

### Influence of orientation of expression cassettes on transgene expression

**Figure 4:**
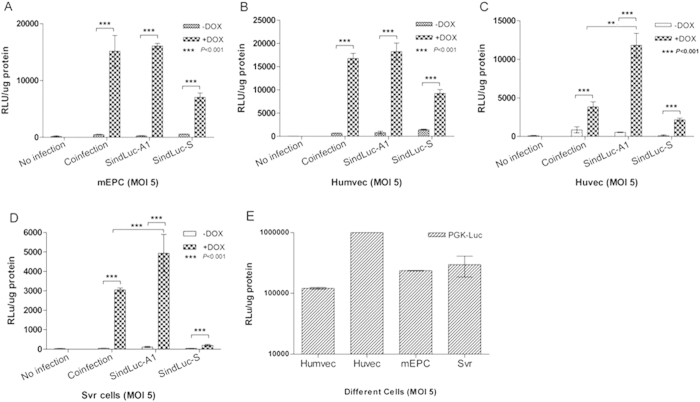
Quantification of doxycycline-dependent gene expression in endothelial cells infected with different lentiviral vectors carrying luciferase transgene. Different cell lines transduced with various vectors (dose: MOI 5) were cultured in the presence or absence of Dox (4 μg/mL) and subjected to luciferase activity measurement at 96 hours post transduction. Coinfection: TRELuc and VEcadrtTA vectors were used. (**A**) Luciferase detection in murine endothelial progenitor cells (mEPC). (**B**) Luciferase detection in human microvascular endothelial cells (Humvec). (**C**) Luciferase detection in human umbilical vein endothelial cells (Huvec). (**D**) Luciferase detection in SVR cells. (**E**) Luciferase detection in different cell lines infected with PGK-Luc vector as a positive control. (***P < 0.001). Results are presented as mean ± SD of data from each group, n = 3.

Different endothelial cell lines were transduced with the Tet-inducible lentiviral vectors (MOI 5) in the presence or absence of Dox (4 μg/mL). Luciferase expression was measured at 96 hours post-transduction. We detected a low luciferase expression in all inducible vectors in the absence of Dox, indicative of leaky expression in the uninduced state (Fig. 4A–D). A robust Dox-dependent luciferase expression was detected when the rtTA2s-M2 transactivator was present (vector: SindLuc-A1). Placing the two cassettes in the opposite orientations resulted in lower uninduced levels of transgene expression and higher induction efficiency in the presence of the inducer. The PGK-Luc vector was used as a positive control in all the experiments. Lentivirus had the highest infection efficiency in Huvec cells among the tested endothelial cell lines (Fig. 4E). Based on the above data, we considered the SindLuc-A1 as an optimal viral vector because it had the lowest level of leaky expression in the uninduced state and the highest luciferase expression after induction in the selected endothelial cell lines (Fig. 4A–D).

### Expression of the tet-on system driven by endothelial-specific or non-endothelial promoters

**Figure 5:**
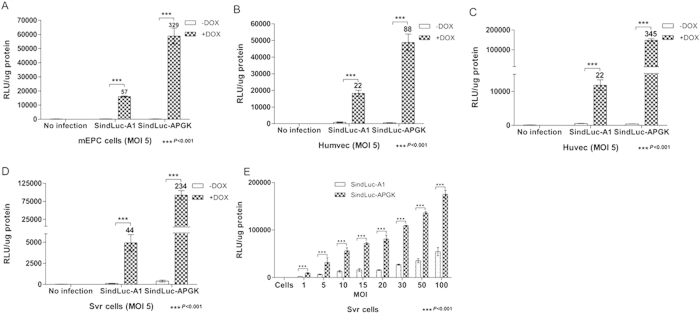
Quantification of doxycycline-dependent gene expression in endothelial cells infected with different lentiviral vectors carrying luciferase transgene. Different cell lines transduced with SindLuc-A1 or SindLuc-APGK vector (MOI 5) were cultured in the presence or absence of Dox (4 μg/mL) and subjected to luciferase activity measurement at 96 hours post transduction. Fold induction is indicated at the top of each bar. (**A**) Luciferase detection in murine endothelial progenitor cells (mEPC). (**B**) Luciferase detection in human microvascular endothelial cells (Humvec). (**C**) Luciferase detection in human umbilical vein endothelial cells (Huvec). (**D**) Luciferase detection in SVR cells. (**E**) Luciferase detection in SVR cells infected with SindLuc-A1 or SindLuc-APGK vector in a dose-growing scale (MOI 0; 1; 5; 10; 15; 20; 30; 50; and 100). Results are presented as mean ± SD of data from each group, n = 3.

To compare the function of promoters with endothelial-specific or constitutive activity, we generated a new single inducible lentiviral vector with a human phosphoglycerate kinase (hPGK) constitutive promoter that replaced the endothelial specific promoter VEcad used in the SindLuc-A1 construct (vector: SindLuc-APKG, Fig. 1E). In this case, the transactivator (rtTA2S-M2) was driven by the constitutive promoter hPGK. Different endothelial cell lines were transduced with SindLuc-A1 or SindLuc-APGK lentiviral vectors (MOI 5). The cells transduced with single inducible vector, SindLuc-APGK, produced high luciferase expression (~100.000 RLU/μg protein), which was 88- to 345-fold higher in the presence of Dox (4 μg/mL) when compared to the basal expression (<800 RLU/μg protein) in the absence of Dox. Additionally, we investigated the relation between MOI and luciferase expression level in SVR cells. As expected, increasing the vector dose led to augmented luciferase expression in the presence of Dox (Fig. 5E).

### *In vivo* biological function of single inducible lentiviral vectors

Analysis of the biological function of the single inducible lentiviral vectors (SindLuc-A1 and SindLuc-APGK) was carried out in a gastrointestinal cancer model (MC38, murine colon carcinoma). The MC38 cells were injected subcutaneously into the dorsal area of mice. Intra-tumor injection of different lentiviruses was performed 10 days later. Dox was administrated in drinking water for 10 days and the animals were sacrificed at day 21 of the experiment.

**Figure 6:**
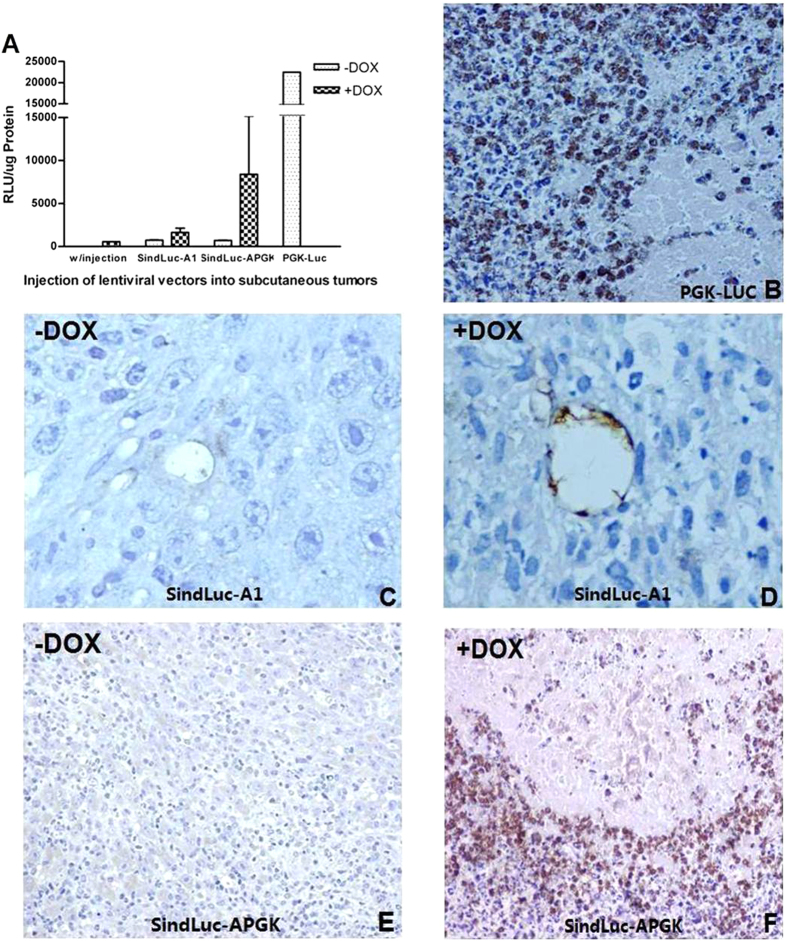
Quantification of luciferase expression *in vivo* and localization of luciferase protein in tumor tissues by immunohistochemistry. (**A**) Luciferase activity measurement in tumors collected from each experimental group. (**B**) A 40× magnification of the tumor treated with unregulable PGK-Luc vector. (**C**) A 200× magnification of the tumor treated with regulable SindLuc-A1 vector without Dox induction. (**D**) A 200× magnification of the tumor treated with regulable SindLuc-A1 vector with Dox induction. (**E**) A 40× magnification of the tumor treated with regulable SindLuc-APGK vector without Dox induction. (**F**) A 40× magnification of the tumor treated with regulable SindLuc-APGK vector with Dox induction. Immunohistochemistry was performed on paraffin-embedded tumor sections. The presence of intracellular luciferase was visualized in brown due to peroxidase reaction. The tissues were counterstained with hematoxylin, which stains the nuclei blue. Results are presented as mean ± SD of data from each group, n = 3.

The transgene expression was evaluated by luciferase activity measurement and *in situ* detection of luciferase protein in tumor tissue. Luciferase activity was highest in those tumors collected from mice treated with unregulable PGK-Luc vector. This group served as a positive control in this experiment (Fig. 6A). The detected luciferase signal in SindLuc-APGK (+Dox) mice was 10- to 22-fold higher than in the uninduced group (Fig. 6A). SindLuc-A1 (+DOX) mice reached two-fold higher expression of luciferase compared to the SindLuc-A1 (-DOX) mice. SindLuc-A1 (-DOX) and SindLuc-APGK (-DOX) mice had nearly undetectable levels of luciferase activity; bioluminescence signal level detected in the tumors of these animals was comparable to that in mice without any injection (negative control). Indeed, such levels of luciferase expression were considered as a background signal (Fig. 6A).

The intra-tumor expression of luciferase detected by immunohistochemistry was consistent with the luciferase activity measured above. We observed that the majority of tumor cells were positive for luciferase expression in the tissue samples collected from animals in PGK-LUC mice (Fig. 6B). Furthermore, we were able to detect the luciferase-positive cells only in the endothelium lining the vessels of the tumor nodules excised from SindLuc-A1 (+DOX) mice (Fig. 6D). However, no luciferase-positive cells were detected in the tumor tissue collected from SindLuc-A1 (-DOX) mice (Fig. 6C). Similarly, the tumors that have been grown in the SindLuc-APGK (-DOX) mice were negative for luciferase expression as well (Fig. 6E). In contrast, ubiquitous luciferase-positive cells were easily detected in tumors isolated from the SindLuc-APGK (+Dox) mice (Fig. 6F). In conclusion, our results demonstrated that the endothelial lineage-specific single regulable lentiviral vector can limit the reporter gene expression in tumor endothelial cells *in vivo* following doxycycline induction.

In summary, we believe that the single inducible lentiviral vector with an endothelial lineage-specific promoter as demonstrated in our study could be used for efficient targeting of endothelium at the sites of tumor angiogenesis. The induced gene expression within the tumor would reach therapeutic levels through this novel cancer treatment strategy.

## Discussion

In a previously published study^21^, an increased dose of inducible vector produced high background expression of a transgene. This could result from the dose-dependent accumulation of the vector genomes integrating into chromosomal locations in the transduced cell populations, where the TRE/min was constitutively activated. It will be necessary, for future clinical trials, to reduce the amount of vectors used to avoid the risk as a result of viral integration into the host genome^16^.

The aim of this study was to develop an endothelial lineage-specific single inducible lentiviral vector that permits doxycycline to control the expression of therapeutic transgenes in endothelial cells. The importance of this project attaches to the possibility it raises of targeting tumor vascular tissue for therapeutic purposes using lentiviral vectors directly or stable endothelial progenitor cell lines, which have been recombined with a single endothelial-specific, Dox-inducible expression of therapeutic genes. Thus, we generated a single lentiviral vector containing both the transgene-inducible expression cassette and the transactivator expression cassette. Compared with other research groups^14^,^19^,^22^,^23^, we generated an optimal single inducible vector construct by using TREalb inducible promoter. Next, we cloned a minimal mono-directional poly (A) site into this structure. Due to limitations of the lentivirus packaging capacity, this minimal poly (A) site could be used widely for other vector designs in the future. Finally, we orientated the transactivator expression cassette in the antisense direction with respect to the vector RNA sequence without affecting the viral production capability.

A two-vector and several single-vector approaches were compared in our study for delivery of the transactivator rtTA2S-M2 and regulable transgene expression cassettes. We obtained reproducible, regulable and efficient transgene expression in endothelial cells. Moreover, in non-endothelial cells, there were nearly undetectable levels of transgene expression. In a previously described study^14^, the tetracycline-regulated CMV promoter (TRE/CMVmini), composed of a tetracycline-responsive element (TRE) and a CMV minimal promoter, was used. Placing the cassettes in the opposite orientation resulted in a slightly higher induction level not only in presence of Dox, but also higher in the uninduced state, compared with placing the cassettes in the same orientation. The major factor responsible for the distinct expression level was the minimal promoter used in those studies, which was also supported by another finding^24^; a tetracycline-regulated albumin promoter produced a lower gene expression in the uninduced state than TRE/CMVmini and could achieve a high induction level^10^. Rather than using an all-in-one vector^14^,^19^,^24^, we chose this tetracycline-regulated albumin-inducible promoter for our study to reduce basal expression levels without using repressor molecules.

During viral production, all the SindLuc-A vectors presented higher titer than the SindLuc-S vector. This result indicated that consistently oriented transcriptional products, as observed in the case of the SindLuc-S vector, can reduce the yield of viral mRNA. Both transcription products were terminated by the same Poly (A) signal which was located in the R region of 3′ LTR. In addition, in the set of SindLuc-A vectors, there are no any transcriptional products interfering with viral mRNA composition during the viral production process.

There was a very low transgene expression level in the uninduced state in all of our experiments (MOI 5–20). The action of the vectors was different depending on the relative orientation of the transgene and rtTA2S-M2 transactivator cassettes. Additionally, the same inducible vector possesses the different induction level in various endothelial cell lines. This might be attributed to a difference in the activity of the VEcad endothelial promoter in different endothelial cells (Fig. 2A–D). From all of the vectors tested, we found SindLuc-A1 to be optimal because transgene expression was absent in the state of Dox deficiency and was induced around 80-fold when Dox (1 μg/mL) was added to the medium. These data indicate that there was no influence between the two promoters (VEcadh/TREalb). Based on the same structure, a non-lineage-specific single inducible vector was constructed by replacing an endothelial specific VEcad promoter with an hPGK promoter (vector: SindLuc-APGK). In the cells transduced with SindLuc-APGK at MOI 5, luciferase expression was undetectable in the absence of Dox, and there was a very high induction efficiency (~345-fold) in the presence of Dox (Fig. 5A–C). Likewise, the maximal luciferase expression level obtained with this vector was only 10 times lower than that achieved with PGK-Luc unregulable vector (Fig. 4E). Furthermore, we also generated a vector, SindLuc-A2, which contained a 2.9-Kb stuffer DNA between the two promoters; a longer fragment was not allowed due to the maximal packaging capacity (8–9 K bps) of lentiviral vector. It seemed that the 2.9-Kb stuffer DNA had a negative influence on Dox-dependent induction (Fig. 2A–D). This stuffer DNA sequence plays an unknown role in the function of this vector. However, in the viral production process, the titer of this particular virus was similar to the titer of SindLuc-A1 vector (Table 1).

In our study, when the two expression cassettes were placed in the same orientation (vector: SindLuc-S) with respect to viral RNA sequence, it seemed that the transgene expression was affected in the doxycycline induced state. Previous studies also indicated that the same orientation of cassettes in a vector affected transcription when using the Tet-responsive promoter^25^,^26^. Although this effect resulted in the suppression of its basal activity, it strongly reduced the expression of transgenes in the “on” state^17^. In another study^14^, a similar result was also reported; the same orientation of cassettes in a vector involved overlapping expression cassettes. The cassette was required for driving the production of the genome of the vector from the precursor plasmid, and the cassettes used to express the transactivator and the transgene share the same poly (A) sequence, which is in the 3′LTR of the vector. In our study, we found that the action of the SindLuc-S vector was not satisfactory in the Dox-induction state. It produced only around 4-to 30-fold induction with Dox, and the maximal transgene expression level was much lower than that from the SindLuc-A1 vector (Fig. 2A–D).

In our work, we tested the kinetics of Dox induction and harvesting time in relation to induced gene expression by adding the Dox (4 μg/mL) to the culture medium at 4, 24 or 48 hours after infection and harvesting cells for transgene activity measurement at 72 or 96 hours post transduction. The highest luciferase expression was observed, when the Dox was added at 48 hours (Fig. 3B), which is higher than previously published results^14^,^20^. Compared to a previously described lentiviral infection protocol^27^, in which luciferase activity was measured at 72 hours post transduction, our induction protocol, in which we kept the cells for 96 hours in the presence of Dox, achieved higher transgene expression. Thus, we have demonstrated that we could increase the luciferase expression by 2- to 3- fold with our newly established protocol, compared to previously described method^27^. For optimal doxycycline induction, we found that cells should be infected with inducible vectors during the first 48 hours and cultured for the subsequent 48 hours in the presence of Dox at 4 μg/mL. The cells are then harvested for transgene detection after 96 hours of culturing.

In distinction from previous studies^14^,^17^, we developed a minimal monodirectional poly (A) sequence for a single inducible vector. Lentiviral vectors have a less-than-8-Kb site for foreign cassette insertion. Our intent was to minimize the vector size without affecting its function in order to reduce the genome integration sequence. It is also a new design of a minimal lentiviral vector. In a previous study^28^, researchers generated a minimal Rev-Response Element and packaging signals for HIV virus. In future clinical trials, a minimal lentiviral vector will be required to avoid the production of replicatively competent recombinants—one that can be modified by reducing the congenetic sequence from wide-type HIV virus. This minimal synthetic monodirectional poly (A) site should be very useful for the novel vector designs.

We have chosen the murine vascular endothelial cadherin promoter (VEcad) for our study because of its highly specific activity in endothelial cells with no reported activity in non-endothelial cells. In 2003, Dancer *et al.* used VEcad promoter to drive the expression of thymidine kinase and successfully inhibit tumor growth of Lewis lung carcinoma cells in transgenic mice^29^. In 2005, the human VEcad promoter transcriptional activity was also characterized, and the hVEcad promoter was subjected to organ-specific regulation and was activated in tumor angiogenesis^30^,^31^. The inducible endothelial-specific lentiviral system we have developed is expected to have a broad application in future tumor therapy. For tumors that are easily accessible, such as in melanoma or head and neck tumors, the lentiviruses could be injected directly into the tumor mass. However, for internal tumors the lentiviruses could be used to transduce isolated EPCs from patients *ex vivo* and then the transduced EPCs can be infused to patients. We believe our novel lineage-specific single inducible lentiviral vector will provide a new and effective resource in the future treatment of human tumors.

## Methods

### General methods

All methods described in this manuscript were carried out in accordance with the approved guidelines in the University of Navarra, Pamplona, Spain.

### Animals and cell lines

Five- to six-week-old female C57BL76J mice were purchased from Harlan Laboratories (Barcelona, Spain). Animals were maintained under standard conditions and all procedures were approved by the institutional ethical committee. The viral vector was administered by intratumoral injection of 70 μL of saline to evaluate its biological function in the Tet-on system. Doxycycline (Dox) induction started 48 hours after the vector injection. Dox (Sigma-Aldrich, St Louis, USA) was given in drinking water (1 mg/mL with 10% sucrose). The following cell lines were obtained from the American Type Culture Collection: human embryonic kidney 293 (HEK293) cells, human embryonic kidney 293 T (HEK293T) cells, human cervical carcinoma (HeLa), murine colon carcinoma (MC38), human angiosarcoma (SVR), human umbilical vein endothelial cells (Huvec) and human microvascular endothelial cells (Humvec). Murine endothelial progenitor cells (mEPC) were obtained from animals as primary culture cells.

The cell lines were cultured in an incubator at 37 °C with 5% CO_2_ and various media. Specifically, HEK293, HEK293T, HeLa, and MC38 cells were cultured in standard DMEM supplemented with 10% fetal bovine serum (FBS), 100 IU/mL penicillin, 100 μg/mL streptomycin and 2 mM glutamine (all from Life Technologies, Carlsbad, CA). Huvec cells were cultured in M131 medium (Life Technologies) supplemented with 25 mM HEPES (Life Technologies), 10% FBS, 100 IU/mL penicillin, 100 μg/mL streptomycin, 2 mM glutamine, 50 μg/mL heparin (Mayne Pharma, Madrid, Spain) and 25 μg/mL of Endothelial Cell Growth Supplement (Sigma). Cells were cultured on human fibronectin (Sigma) coated 75- or 125-cm^2^ flasks and plates (Sarstedt, Wexford, Ireland). Humvec were cultured in M131 medium supplemented with 5% (v/v) Microvascular Endothelial Cells Growth Supplement (MVGS, Life Technologies), 10% FBS, 100 IU/mL penicillin, 100 μg/mL streptomycin, 2 mM glutamine and 50 μg/mL heparin. Cells were cultured on fibronectin-coated flasks and plates.

EPCs were cultured according to the protocol as previously described^32^. Briefly, bone marrow-derived mononuclear cells (MNC) were isolated by density gradient centrifugation with Ficoll Histopaque (Sigma). After purification, 8 × 10^6^ MNCs were plated on fibronectin-coated six-well plates (Cellstar, Greiner *Bio-One, Frickenhausen*, Germany). Cells were cultured in EBM-2 medium (Clonetics, Walkersville, MD) with supplements: 10 ng/mL recombinant rat VEGF, 1 ng/mL bovine basic FGF, 10 ng/mL recombinant mouse IGF-I, 10 ng/mL recombinant human EGF (all from R&D Systems, Minneapolis, MN), 1 μg/mL Hydrocortisone (Sigma) and 5% FBS. The cultured cells were replated on day 4 and used for particular experiments.

### Construction of INDUCIBLE LENTIVIRAL VECTORS

The third generation self-inactivating and inducible lentiviral vectors were generated on the basis of the backbone pRRL.cPPT.PGK.eGFP.WPRE as described^23^. A *BamH1-SaL1* digest of the synthetic DNA linker containing Poly-cloning sites (*BamH1-Xho1-Xma1-Sma1-Xba1-Mlu1-Nhe1-BglII-SaL1*) was performed for the ligation into a *BamH1-SaL1* digest of pRRL.cPPT.PGK.eGFP.WPRE to obtain the particular construct of pRRL.cPPT.PGK.linker.WPRE. The firefly luciferase was released from pGl3-basic by *Xma1-Xba1* digestion and was ligated into *Xma1-Xba1* digested pRRL.cPPT.PGK.linker.WPRE to obtain a lentiviral vector encoding PGK-Luc. The PGK promoter from pRRL.cPPT.PGK.linker.WPRE was removed by *Xho1* digestion to get pRRL.cPPT.linker.WPRE. The *Nhe1-BamH1* digestion of pmVEcad-rtTA (No PA) released the VEcad-rtTA fragment for subsequent ligation into an *Nhe1-BglII* digest of pRRL.cPPT.Linker.WPRE to achieve the lentiviral vector expressing the mVEcadrtTA fragment. TREalb-Luc sequence was isolated from pTREalb-Luc with *Xho1-Xba1* and placed into the *Xho1-Xba1* digested pRRL.cPPT.linker.WPRE to create the TREalb-Luc lentiviral vector. The S60PA element is derived from published result^33^ with slight modifications. The sequence of our modified S60PA is 5′-GATCCAATAAAAGATCTTAAGTTTCATT AGATCTGTGTGTTGGTTTTTTGTGTG-3′, where the two underlined nucleotides were mutated to prevent the transcriptional stop function of the polyA. To construct SindLuc-A1 lentiviral vector, the mVEcad-rtTA-S60PA cassette was excised with *Xho1-Acc1*, and digestion of pmVEcad-rtTA (S60 PA) was done for the ligation into the pTRELuc vector with *Xho1-ClaI*. A 2.9 Kbp stuffer DNA fragment was excised from STK120 with *Xho1* digestion and was ligated into *Xho1* digest site of SindLuc-A1 to gain the SindLuc-A2 lentiviral vector. To construct the SindLuc-S lentiviral vector, the mVEcad-rtTA fragment was released with *Nhe1-BamH1* from pmVEcad-rtTA (No PA) and finally ligated into TRELuc with *Nhe1-BglII*. Digestion of pUHrT62-1 with *BamH1-EcoR1* (blunted) excised the rtTA2S-M2 fragment that was linked into pRRL.cPPT.PGK.eGFP.WPRE with *BamH1-Sal1* (blunted) to obtain the pRRL.cPPT.PGK.rtTA.WPRE construct. The PGK-rtTA (half gene) fragment was generated from pRRL.cPPT.PGK.eGFP.WPRE with *Xho1-Pml1* digestion and was ligated into a pmVEcad-rtTA (S60 PA) plasmid, digested with *Xho1-Pml1* to acquire phPGK-rtTA (S60 PA). SindLuc-APGK lentiviral vector was obtained by excision of the hPGK-rtTA-60PA expression cassette from the phPGK-rtTA (S60 PA) plasmid with *Xho1-Acc1* digestion and ligated into a TRELuc lentiviral vector with *Xho1-Cla1*.

### Lentiviral vector production

Dr Antonia Follenzi (University of Torino Medical School, Italy) kindly provided all the plasmids for lentiviral vector production. Vectors were produced by transfection of HEK293T cells as described^34^, with the following modifications. HEK293T cells (7 × 10^6^/plate) were growing in 15-cm-diameter dishes for 24 hours prior to transfection in DMEM supplemented with 10% FBS, penicillin (100 IU/mL) and streptomycin (100 μg/mL) at 37 °C in a 5% CO_2_ incubator. The culture medium was exchanged 2 hours before transfection (DMEM with 2% FBS). Transient transfection of HEK293T cells with the plasmids was obtained by the calcium phosphate precipitation method. Briefly, a total of 40 μg of plasmid DNA was used for the transfection of one culture dish, including 12 μg of the envelope plasmid (pMD.G) encoding VSV-G, 5 μg of the packaging plasmid (pMDLg/pRRE), 3 μg of the plasmid producing Rev regulatory protein (pRSVrev) and 20 μg of the transfer vector plasmid. The calcium phosphate precipitate was formed by adding the plasmids to a final volume of 1000 μl of filtered dH_2_O and 200 μl of 2.5 M CaCl_2_ solution. Next, 1000 μl of 2×-concentrated HEPES-buffered saline (281 mM NaCl, 100 mM HEPES, 1.5 mM Na_2_HPO_4_, pH 7,05) was added drop-wise with brief vortexing for 10 seconds. After 15 minutes, the precipitate was added to cell cultures. Post-transfection culture medium (16 hours) was replaced with DMEM (10% FBS). The conditioned medium was collected after 24 hours, cleared by low-speed centrifugation and filtered through a 0.45-μm-pore filter (500 mL) (Dominique *Dutscher* S.A., Brumath, France). All the vectors were concentrated by ultracentrifugation at 19,500 rpm for 2 hours at 12 °C, aliquoted, and stored at −80 °C until use.

### Lentiviral vector titration

Infectious viral particles were determined by transduction of 5 × 10^4^ HeLa cells with serial dilutions of the vector preparation in a 24-well plate in the presence of 8 μg/mL Polybrene (Sigma). 72 hours later, genomic DNA from transduced HeLa cells was extracted using a DNA Blood Mini Kit 50 (Qiagen, Santa Clara, CA). The transducing unit titer (TU/mL) was determined by quantitative PCR (qPCR) as described^35^. Real-time PCR was used for the quantitative analysis of proviral DNA copies. Probes were labeled at the 5′end with the reporter dye molecule FAM (emission wavelength: 518 nm) and at the 3′end with the quencher dye TAMRA (emission wavelength: 582 nm). The 3′end of the probe was additionally phosphorylated to prevent extension during PCR. For detection of the lentivirus WPRE sequence in HeLa cells, the following primers and probe were used: forward primer (1277 F): 5′-CCGTTTCAGGCAACGTG-3′; reverse primer (1361 R): 5′-AGCTGACAGGTGGTGGCAAT-3′; probe (1314 P): 5′-FAM-TGCTGACGCAACCCCCACTGGT-TAMRA-3′ [52]. For human β-actin gene copy numbers in HeLa cells, the following primers and probe were used: forward primer, 5′-GCGAGAAGATGACCCAGCTC-3′; reverse primer: 5′-CCAGTGGTACGGCCAGAGG-3′; probe: 5′-FAM-CCAGCCATGTACGTTGCTATCCAGGC-TAMRA-3′ [53]. For the PCR reaction, the universal PCR Master Mix (4 μL; Promega, Madison, WI), with a 1-μM concentration of each primer, and the probe were combined. Finally, genomic DNA was added to each reaction, and the total reaction volume was adjusted to 10 μL. Standard conditions were used for the PCR reaction (2 minutes at 50 °C, 10 minutes at 95 °C, and then 40 cycles of 15 seconds at 95 °C and 1 minute at 60 °C).

### Cell transduction

Cells (HEK293, HEK293T, HeLa and SVR cell lines) growing in 24-well plates to 50–60% confluence were transduced with lentiviral vector preparations in a total volume of 300 μL of DMEM (10% FBS) supplemented with Polybrene (8 μg/mL). After 16 hours, the cells were washed extensively with PBS to remove lentiviral genomic RNA and fresh medium was added. The cells were maintained in culture for another 72 hours. Other cell lines were incubated with lentiviral vector preparations in a total volume of 300 μL of specific medium (according to the each endothelial cell type) supplemented with Polybrene (8 μg/mL). For inducible lentiviral vectors, various Dox dosages and induction times were tested to optimize the induction conditions.

### Cell transfection

The cells were cotransfected with 1 μg of each transfer plasmid and 20 ng of pRL-SV40 plasmid (Promega). The Huvec and Humvec cells were transfected with Lipofectamine Plus reagent (*Life* Technologies). HEK293 and HEK293T were transfected with polyethylenimine (Polysciences, Warrington, PA). SVR cells were transfected with FuGENE HD transfection reagent (Promega). Finally, HeLa cells were transfected by the Calcium phosphate method. Cells were cultured in 24-well plates for transfection. The post-transfection medium was exchanged according to the protocol for each transfection reagent. The cells were incubated for 48 hours in the presence or absence of Dox and then harvested for the luciferase activity measurement.

### Luciferase detection *in vitro*

The cells transduced with the lentiviral vector were measured with the Dual-Luciferase Reporter Assay System (Promega) for luciferase expression. Following the manufacturer’s instructions, the cells were recovered at different time points, washed twice with PBS, and lysed in 250 μL Passive Lysis Buffer (Promega) by 10 minutes of shaking. The cell extracts were serially diluted, and 20 μL of each dilution were mixed with 100 μL of the luciferase assay reagent in luminometer tubes. The firefly luciferase activity was measured using a single tube luminometer (*Berthold* Detection Systems, Pforzheim, Germany). Protein concentration was calculated using the Bio-Rad protein assay (Bio-Rad, Hercules, CA). Experiments were repeated three times.

### Animal model of gastrointestinal cancer

For the gastrointestinal cancer model, 1 × 10^6^ of MC38 cells (murine colon carcinoma) in 100 μL of saline were injected subcutaneously into the dorsal area of mice. Ten days after the tumor cell inoculation, the tumor-bearing mice were assigned to five groups of three *animals each.* Intratumoral injections of different lentiviral vectors (5 × 10^7^ TU/mL/tumor) were performed. Dox was administrated in drinking water for a period of 10 days. Group A remained unmedicated and served as a negative control group. Group B was injected with SindLuc-A1 vector and drank water without Dox. Group C was injected with SindLuc-A1 vector and drank water treated with Dox. Group D was injected with SindLuc-APGK vector and drank water without Dox. Group E was injected with SindLuc-APGK vector and drank water treated with Dox. Group F was injected with PGK-Luc vector and drank water without Dox. The survival of the animals was checked daily. Finally, the animals were sacrificed by cervical dislocation at day 21 of the experiment. The subcutaneous tumors were collected, and each one was divided into two parts, one part fixed in 4% paraformaldehyde for histological examination and the other part frozen and stored at −80 °C for luciferase activity measurement.

### Immunohistochemistry

Three-μm-thick sections of 4% paraformaldehyde-fixed and paraffin-embedded tumor tissues were processed for immunohistochemistry. Staining with the following antibodies was performed: goat anti-firefly luciferase (1:50; Cortex Biochemical, San Leandro, CA, CR2029GAP), polyclonal rabbit anti-goat immunoglobulins biotinylated antibody (1:600; Dako, Glostrup, Denmark, E0466) and streptavidin- biotinylated horseradish peroxidase (1:100; Amersham, RPN1051V). For detection, a diaminobenzidine reagent (Dako) was used and counterstaining performed with Mayer’s hematoxylin solution (Merck-KGaA, Darmstadt, Germany). Slices were mounted in DePex Mounting Solution. Histological samples were visualized with a Nikon microscope, and images were acquired using a Leica camera (Leica Microsystems, Wetzlar, Germany) and analyzed with Aquacosmos acquisition software (Hamamatsu Photonics, Hamamatsu City, Japan).

### Luciferase detection in tumor tissue

Tumor tissue was cut and lysed with 250 μL of Passive Lysis Buffer (Promega) and homogenized in Eppendorf tubes using the manual method with plastic sticks. The tissue extracts were collected and processed according to the protocol described above in the section on luciferase detection *in vitro*.

### Statistical analysis

All analyses were done using SPSS version 9.0 software (Chicago, IL) with P < 0.05 considered to be statistically significant. Data were analyzed by Mann-Whitney nonparametric tests due to the sample size being less than 10.

## Additional Information

**How to cite this article**: Yang, G. *et al.* Development of Endothelial-Specific Single Inducible Lentiviral Vectors for Genetic Engineering of Endothelial Progenitor Cells. *Sci. Rep.* **5**, 17166; doi: 10.1038/srep17166 (2015).

## Figures and Tables

**Table 1:** The lentiviral titers from different vectors.

Constructed Lentiviral vectors	Final Titer (TU/mL)	Size of Inserted Fragment (bps)
TRELuc	7.3 × 10^8^	1900
VEcadrtTA	8.1 × 10^8^	1075
SindLuc-A1	4.0 × 10^8^	3428
SindLuc-A2	5.3 × 10^8^	6129
SindLuc-APGK	3.8 × 10^8^	3642
SindLuc-S	2.6 × 10^8^	3387
PGK-Luc	7.3 × 10^8^	2312

All the production and titration steps were performed at the same time. The production was completed in 5 15-cm-diameter dishes, the supernatants were harvested at 48 hours, 72 hours, and 96 hours. Titration was performed by qPCR. Each vector production was repeated three times.
